# Nasal vaccine or monoclonal therapy: Which is winning weapon against SARS-CoV-2 variants in 2022?

**DOI:** 10.7189/jogh.12.03023

**Published:** 2022-05-14

**Authors:** Lorenzo Lo Muzio, Francesca Spirito

**Affiliations:** Department of Clinical and Experimental Medicine, University of Foggia, Foggia, Italy

In January 2021, we wrote a letter to the editor of the *Journal of Global Antimicrobial Resistance* about the possible role of vaccine or monoclonal therapy in the fight against SARS-CoV-2 [1]. The virus had already been infecting the world for 12 months and the first variants were emerging, such as the UK variant, B.1.1.7, characterized by a large number of mutations, and the South Africa variant, B.1.351, which emerged independently of B.1.1.7.

All our hopes were placed on vaccines, such as BioNTech/Pfizer, Oxford/AstraZeneca, Moderna, Sinovac, CanSino, Sinopharm, Novavax, and others [1]. However, the first debates had already begun on the usefulness of these vaccinations as new variants were emerging, raising the possibility of infection for recovered/vaccinated subjects. Some reports outlined cases of SARS-CoV-2 reinfection from different countries, including the USA, Ecuador, Hong Kong, and Belgium [2]. The researchers argued on the necessity of verifying whether all these cases were truly reinfection because the possibility of reinfection could drastically reduce the effectiveness of the vaccination campaign and affect government and public-health policy decisions. Cases of COVID-19 were subsequently described in subjects treated with two, or even three doses of vaccine [3], causing doubts in part of the population about the usefulness of vaccination for SARS-CoV-2 and the formation of No-Vax groups.

In the meantime, however, the virus has shown great mutational capacity, so now we are talking about variants Alpha, Beta, Gamma, Delta, Epsilon, Iota, Kappa, Lambda, Mu [4]. This COVID-19 alphabet shows that, in two years of travelling around the planet, the virus is accumulating one new variant after another. The variants are not becoming increasingly dangerous – indeed, most of the coronavirus transformations are harmless. The Alpha variant had already distinguished itself for the greater speed of contagion compared to the original SARS-CoV-2 virus; the Beta affected younger subjects, between 10 and 30 years; Gamma and Epsilon made the already recovered sick again. Lambda has been found to be particularly infectious [5].

In the evolutionary dance for survival, mutations emerge periodically and result in a turbo-charged virus. The Delta, born in India, has this enhanced “engine” that allows it to be transmitted in a few seconds [6]. Will it be the last ace up the sleeve of the SARS-CoV-2, or is it a taste of what awaits us in the future? Are these variants biological disguises that will deceive vaccines and bring us back to the days of intensive care units crowded with gasping patients?

Of all the variants, Mu, first identified in Colombia in January 2021 and now identified in at least 39 countries, appears to be the most worrying, as it has a similar transmission speed to the Delta and is enhanced by other mutations, which could, in theory, allow it greater resistance to being neutralized by antibodies [4].

At the moment, the strategy of the virus seems not to be trying to escape the antibodies but running faster, transmitting itself with particular ability in aerosols and with a shorter incubation period than previous variant of the virus.

However, with a growing vaccinated population, the virus could modify its strategy by selecting mutations that are able to escape vaccinal immunity. The main drug companies are modifying their vaccines to make them more efficacious against the Delta variant. This process could take months, however, and there is a risk that these new vaccines could be overcome by even newer variants.

## SPECIAL PATIENTS AND VACCINES

An unprecedented problem that has emerged in the fight against the SARS-CoV-2 pandemic is the use of nucleic acid-based vaccines in fragile patients, such as those undergoing solid organ transplantation (SOTRecipients). These individuals are at greatest risk of various infectious diseases, and vaccinations are among the most efficient available interventions for inducing effective immunizations. There are currently no consolidated scientific data to support the safety and efficacy of nucleic acid-based vaccines (DNA, RNA) in organ transplant recipients. Solid organ transplant patients are at high risk of poor outcomes with COVID-19. In fact, a recent European study found a 20% mortality in liver transplant (LT) recipients with SARS-CoV-2 infection, markedly higher than in the general population, highlighting the potential benefits of vaccination in these recipients [7]. In addition, the safety and immunogenicity of SARS-CoV-2 mRNA/DNA vaccines have never been clinically tested previously in SOTR. Public health guidelines prioritized SOTR for vaccination as high-risk populations, but more data are required about the real potentiality of vaccines against SARS-CoV-2 in these patients, such as the level of immune response, the efficacy of the immune response, and the best choice of vaccine.

## NEW WEAPONS IN THE FIGHT AGAINST SARS-COV-2

In light of all these considerations, chasing the virus might not be the winning strategy and it should not give us too much confidence. An advisable strategy would be to start producing other types of vaccines in the form of a spray with the objective to induce local immunity in the respiratory mucosa, as already exists for the flu, blocking the entrance door of the virus. Ohtsuka et al. [8] developed an intranasal vaccine against SARS-CoV-2 using the replication-incompetent human parainfluenza virus type 2 (hPIV2) vector BC-PIV. This vaccine can deliver an ectopic gene as stable RNA and an ectopic protein on the envelope that is able to induce high levels of neutralizing IgG and mucosal IgA antibodies in mice, against the spike protein. University of Oxford researchers are now conducting an open-label clinical trial of the intranasal vaccine in healthy human volunteers [9].

**Figure:**
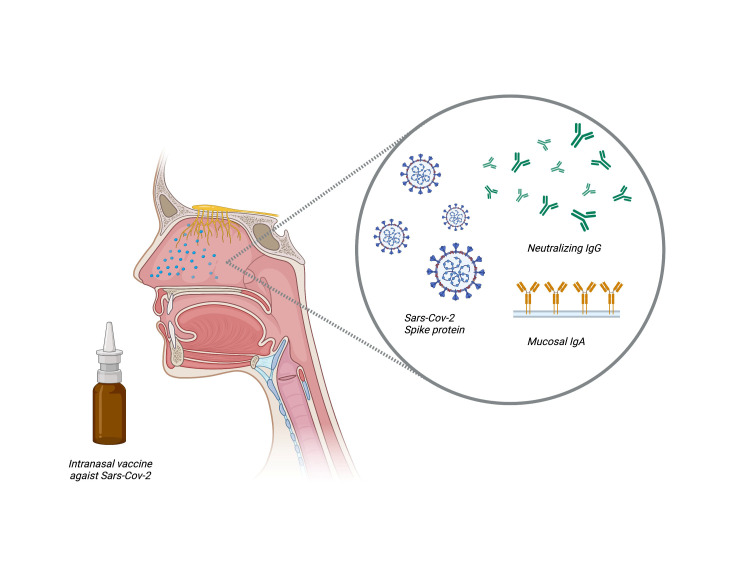
Photo: Intranasal vaccine against SARS-CoV-2. Source: from BioRender (license granted to author).

De Vries et al. [10] designed lipopeptide fusion inhibitors that block the critical first step of infection – the membrane fusion between the viral and host cell membranes – which is mediated by the viral spike protein. Daily intranasal administration to ferrets completely prevented SARS-CoV-2 direct-contact transmission during 24-hour cohousing with infected animals, under stringent conditions that resulted in infection of 100% of untreated animals [10]. These lipopeptides are highly stable and thus may readily translate into safe and effective intranasal prophylaxis to reduce transmission of SARS-CoV-2 [10].

Another possible strategy is to use innovative treatment alternatives such as monoclonal antibodies that could offer short-term protection to those who are not yet vaccinated or who lack a proper response to vaccination, such as immunocompromised patients. Additionally, mAbs could prove helpful during times when circulating variant viruses are not adequately covered by vaccines protection [11]. Moreover, because a certain time is required after vaccination to develop a proper immune response, the benefits of passive immunization are evident in numerous settings where outbreaks are frequent. Some studies are evaluating the potential role of mAbs for prevention of infection or symptomatic disease, such as the phase 3 BLAZE-2 trial (NCT04497907) designed to evaluate the efficacy and safety of bamlanivimab (4200 mg, iv), the Part A of the Regeneron trial (NCT04452318) designed to assess the efficacy and safety of the subcutaneous administration of casirivimab plus imdevimab (600/600 mg) in preventing SARS-CoV-2 infection, the PROVENT trial (NCT04625725) and the STORM CHASER trial (NCT04625972), designed to study the combination of two long-acting antibodies (cilgavimab + tixagevimab), developed from the B-cells of a convalescent donor after the infection [12].

In order to convince the most fearful subjects, some researchers are examining the possibility of mAbs administration via nasal sprays or aerosolized formulations, and early clinical studies confirmed that this approach is safe and can be used to prevent and treat SARS-CoV-2 infection.

Other alternative possibilities in the fight against SARS-CoV-2 could be nanotechnology applications such as viral inactivators (including cellular nanosponges) for reducing viral adhesion, or the treatment of personal protective equipment (PPE) with charged metallic (such as Cu, Ag, Fe and Zn among others) nanoparticles, which seems to result in the release of antiviral agents (ie, reactive oxygen species) which are able to inhibit viral entry into host cells by means of interactions with cell receptors [13].

Nanotechnology offers a way to improve the safety offered by personal protective equipment by modifying their surface, ensuring not only the capture and inactivation of viruses, but also the reusability and washability, without compromising the efficiency and safety [14].

## CONCLUSIONS

In conclusion, because we are lacking standardized means to predict guaranteed immunity in 100% of recipients, it is necessary to continue to develop alternative strategies to vaccines in the fight against SARS-CoV-2.
